# Epigoitrin, an Alkaloid From *Isatis indigotica*, Reduces H1N1 Infection in Stress-Induced Susceptible Model *in vivo* and *in vitro*

**DOI:** 10.3389/fphar.2019.00078

**Published:** 2019-02-07

**Authors:** Zhuo Luo, Li-Fang Liu, Xiao-Hua Wang, Wen Li, Chong Jie, Huan Chen, Fan-Qin Wei, Dan-Hua Lu, Chang-Yu Yan, Bo Liu, Hiroshi Kurihara, Yi-Fang Li, Rong-Rong He

**Affiliations:** ^1^Guangdong Engineering Research Center of Chinese Medicine & Disease Susceptibility, Jinan University, Guangzhou, China; ^2^Guangdong Province Key Laboratory of Pharmacodynamic Constituents of TCM and New Drugs Research, College of Pharmacy, Jinan University, Guangzhou, China; ^3^Department of Otorhinolaryngology, Head and Neck Surgery, The First Affiliated Hospital, Sun Yat-sen University, Guangzhou, China; ^4^State Key Laboratory of Biotherapy, West China Hospital, Sichuan University, Chengdu, China

**Keywords:** epigoitrin, influenza virus, stress-induced susceptibility, MFN2, MAVS

## Abstract

Stress has been proven to modulate an individual’s immune system through the release of pituitary and adrenal hormones such as the catecholamines, growth hormone, and glucocorticoids. These signal molecules can significantly alter the host immune system and make it susceptible to viral infection. In this study, we investigate whether epigoitrin, a natural alkaloid from *Isatis indigotica*, provides protection against influenza infection by reducing the host’s susceptibility to influenza virus under stress and its underlying mechanism. To support it, the mouse restraint stress model and the corticosterone-induced stress model were employed. Our results demonstrated that epigoitrin significantly decreased the susceptibility of restraint mice to influenza virus, evidenced by lowered mortality, attenuated inflammation, and decreased viral replications in lungs. Further results revealed that epigoitrin reduced the protein expression of mitofusin-2 (MFN2), which elevated mitochondria antiviral signaling (MAVS) protein expression and subsequently increased the production of IFN-β and interferon inducible transmembrane 3 (IFITM3), thereby helping to fight viral infections. In conclusion, our study indicated that epigoitrin could reduce the susceptibility to influenza virus via mitochondrial antiviral signaling.

## Introduction

Influenza A viruses (IAVs), highly contagious pathogens, are responsible for severe respiratory infection in humans and animals worldwide with pandemic potential (Chen et al., 2018). At present, antiviral drugs and vaccines are the main treatment for IAVs infection. Due to the high mutation rate and antiviral-drug-resistant strains of IAVs (Kumar et al., 2018), developing vaccines and anti-viral drugs for IAVs infection are still full of challenges. Hence, there is an urgent need to identify novel antiviral therapies or complementary strategies.

Many herbal extracts or natural products have been demonstrated to possess potent anti-influenza, preventive and immunomodulatory effects. The dry root of *Isatis indigotica* (Ban Lan Gen, BLG), a traditional Chinese medicine, has been used for anti-influenza in clinics over thousands of years in China (Zhou and Zhang, 2013). Chemical studies showed that BLG contains various compounds such as alkaloids, nucleosides, amino acids, organic acids (Xiao et al., 2014). Epigoitrin as an alkaloid was used as a marker compound of BLG in the 2015 edition of the Chinese Pharmacopoeia (National Pharmacopoeia Commission, 2015). It has previously been reported that epigoitrin exerts antiviral activity against influenza A1 virus FM1 via inhibiting virus attachment and multiplication *in vitro* (Xiao et al., 2016). However, no *in vivo* pharmacological studies confirmed the anti-influenza activities. Our previous studies indicated that restraint stress could increase the susceptibility to the influenza virus in mice and provide a useful model basis for evaluating the effectiveness of the herbal medicinal product and natural products (He et al., 2011; Tang et al., 2014; Chen et al., 2017). It is well known that stressful events take a toll in the development of disease, especially in infectious disease. Stressors can increase susceptibility to infectious agents, dysregulate the humoral and cellular immune responses to pathogens and increase the risk of catching infectious diseases. Restraint is a commonly used stressor for mice. Mice are placed in tubes with holes such that they can breathe and move forward or backward but cannot turn around, which is often applied overnight during the most active time for mice (Glaser and Kiecolt-Glaser, 2005). Moreover, influenza and pneumonia are the fifth leading cause of death among individuals over 50 years old, which was related to greater immunological impairments associated with distress or depression in the old than that in the young (Glaser and Kiecolt-Glaser, 2005). Accordingly, stress-related immune disorders may be a core mechanism behind multiple infectious diseases, and if antiviral drugs or compounds have the ability to regulate stress-mediated immune disorders, they might play a more important role in the treatment of influenza. In this study, we employed the restraint-stress induced susceptible model to investigate the preventive effects of epigoitrin on influenza infection and its related mechanisms.

## Materials and Methods

### Compounds

Epigoitrin with 98% purity was purchased from Aladdin Biochemical Technology Co., Ltd. (Shanghai, China). Oseltamivir was obtained from Yichang Changjiang Pharmaceutical Co., Ltd. (Wuhan, China). Corticosterone was purchased from Sigma (MO, United States).

### Virus

The human HlN1 prototype strain, mouse-adapted A/FM/1/47 virus (Smeenk and Brown, 1994), was provided by College of Veterinary Medicine of South China Agricultural University (Guangzhou, China). Viruses were propagated in the allantoic cavities of specific-pathogen-free fertilized eggs. The allantoic fluid containing virus was harvested and stored in aliquots at −80°C until used. Median tissue culture infective dose (TCID_50_) was measured in MDCK cells and calculated according to the Reed-Muench formula after serial dilution of the stock. Amounts of 10 TCID_50_ value were used for viral infection in all the cell experiments.

### Mice and Experimental Design

Specific-pathogen-free male Kunming mice with 4 weeks of age and weighing 12–15 g were purchased from Guangdong Medical Laboratory Animal Center (Guangzhou, China). The animals performed in this study were housed in plastic cages and lived under standard laboratory conditions. Animal experiments were approved by the Animal Care and Use Committee of Jinan University (Approval ID: SYXK 20150310001) and performed in compliance with the National Institute of Health’s Guide for the Care and Use of Laboratory Animals (7th edition, United States).

To evaluate the anti-influenza virus effects of epigoitrin on mice loaded with restraint stress, mice were randomly distributed to six groups: Control, Virus, “Restraint + Virus,” Oseltamivir (30 mg/kg/d oseltamivir + restraint + virus), Epigoitrin-L (88 mg/kg/d epigoitrin + restraint + virus), and Epigoitrin-H (176 mg/kg/d epigoitrin + restraint + virus). Oseltamivir and epigoitrin were administered orally to mice for 7 consecutive days, while other groups were received oral administration of water only. After the first day of administration, mice except those in Control and Virus groups were physically restricted in the plastic centrifuge tube of 50 mL with holes for 22 h. On the second day after restraint, mice were anesthetized by inhalation of diethyl ether vapor and then were inoculated intranasally with 500 PFU Influenza virus in PBS. Subsequently, the daily changes of mice in survival and their typical influenza symptoms, including hunched back, ruffled fur, altered respiration and unresponsiveness, were observed and recorded for 21 days or until death. The morbidity of the mouse was estimated when its weight was decreased over 1 g⋅d^−1^. The survival rate was also calculated.

Mice were weighed and euthanized after 5 days post infection (dpi), and the lungs were removed and weighed. The lung index was calculated according to the formula: Lung index (mg/g) = lung weight/body weight. Samples of lung tissue were reserved for histopathological examination, virus titers, and western blotting analysis.

The second animal experiment was conducted to investigate the effect and mechanism of epigoitrin on type I IFN secretion in stressed mice. Mice were distributed at random to five groups: Control, Virus, “Restraint+Virus,” Epigoitrin-L, and Epigoitrin-H. The following treatment was the same as described above. The lung tissues were collected to determine the protein expressions related with IFN-β and MAVS signaling. To explore the effects of epigoitrin on corticosterone level, Mice were randomly divided into four groups: Virus, “Restraint+H1N1,” Epigoitrin-L and Epigoitrin-H. On the second day after restraint, mice were challenged with virus. Blood samples were collected from the heart to determine the plasma corticosterone levels. For investigating the anti-viral activity against H1N1 in unstressed mice, Mice were orally administered with epigoitrin-L (88 mg/kg/d epigoitrin + virus) and epigoitrin-H (176 mg/kg/d epigoitrin + virus) for 7 days prior to H1N1 infection. After 5 dpi, Lung samples were collected for TCID_50_ assay.

### Histological Analysis of Lung Injury

The lungs of the mice were fixed in 10% neutral buffered formalin and processed routinely. Paraffin sections, 5–10 μm thick, were stained with hematoxylin and eosin and then examined under microscopy in a blinded fashion. Pathological changes were scored based on the criteria (Fang et al., 2011): 0, no pneumonia; 1, mild interstitial pneumonia (<25% of the lung); 2, moderate interstitial pneumonia (25–50% of the lung); 3, severe interstitial pneumonia (>50% of the lung). The sums of scores of different animals were averaged.

### Quantification of Cells and Measurement of Cytokines From Bronchoalveolar Lavage Fluid (BALF)

Mice were anesthetized after 5 dpi and lungs were lavaged by instillation and withdrawal of 1 ml PBS through a tracheal cannula and BAL fluid (BALF) was collected. Total BAL cell numbers were determined using a hemocytometer. After centrifuged at 1500 rpm at 4°C for 5 min, Supernatants were collected to determine the levels of TNF-α and IL-1β using enzyme-linked immunosorbent assay (ELISA) kits (Thermo Fisher Scientific, Waltham, MA, United States) according to the manufacturer’s protocol, and compared with known standards. Cell pellets were resuspended in 200 μl PBS and cellular infiltration was assessed on Wright-Giemsa-stained slides (Nanjing Jiancheng Bioengineering Institute, Nanjing, China).

### Determination of Corticosterone Level in Plasma

Corticosterone was extracted from the plasma and quantified by HPLC as we previously reported (Chen et al., 2017). Briefly, the plasma was collected from blood samples pretreated with 10 μl of heparin after centrifugation at 2500 × *g* for 10 min. Cortisol solution (100 μl, 500 ng/ml) as an internal standard was mixed into plasma (1 ml), and then were extracted by mixing with 2 ml of ethyl acetate thoroughly for three times. The organic phase was collected, washed, and evaporated under nitrogen. The residue was dissolved and analyzed by HPLC with a UV detection at 254 nm (Agilent 1200).

### Cell Culture and Treatment

Human alveolar epithelial cell line (A549) and Madin-Darby canine kidney (MDCK) cells were grown at 37°C in 5% CO_2_ atmosphere in Dulbecco’s modified Eagle’s medium (DMEM; high glucose, with L-glutamine) supplemented with 10% fetal bovine serum (FBS), 100 IU/ml penicillin, and 100 μg/ml streptomycin. For simulating the stress-induced susceptibility to influenza virus infection *in vitro*, corticosterone, an indicator of the stress response, was employed to establish a “Corticosterone + Virus” A549 cell model. Cells were treated with corticosterone (100 μM) for 48 h and then infected with 10 TCID_50_ for 12 h. Based on the “Corticosterone + Virus” model, we then evaluate the antiviral activity of epigoitrin against IAVs. Cells pretreated for 2 h with different concentrations of epigoitrin were challenged with corticosterone for 48 h in the presence of epigoitrin prior to infection, then was infected with H1N1 influenza virus of 10 TCID_50_ for 1.5 h. Twelve hours post infection (hpi), the cells were harvested for RT-qPCR and TCID_50_ assay.

### Viral RNA Quantification by RT-qPCR

Total RNA was extracted using TRIzol reagent (Invitrogen) at indicated time according to manufacturer’s instructions. RNA concentrations were determined by optical density measurement at 260 nm on a spectrophotometer (Thermo Fisher Scientific) and cDNA was synthesized from the purified RNA by both random and oligo (dT) priming using an iScript cDNA synthesis kit (Bio-Rad). Intracellular NP and IFN-β RNA levels were measured using the SYBR green method (Applied Biosystems) on a reverse transcription (RT) machine (CFX Connect^TM^; Applied Biosystems) and the relative values of Actin. The fold induction of viral RNA or innate immune genes over the levels of induction for either mock-infected cells or DMSO-treated control cells was calculated. Primer sequences were as follows: NP forward, 5′-CAGGTACTGGGCCATAAGGAC-3′, and reverse, 5′-GCATTGTCTCCGAAGAAATAAG-3′; IFN-β forward, 5′-CTTACAGGTTACCTCCGAAACTGAA-3′, and reverse, 5′-TTGAAGAATGCTTGAAGCAATTGT-3′; Actin forward, 5′-TGACGTGGACATCCGCAAAG-3′, and reverse, 5′-CTGGAAGGTGGACAGCGAGG-3′.

### TCID_50_ Assay

Briefly, a confluent monolayer of MDCK cells grown in 96-well plates were washed two times with PBS and then inoculated with threefold serial dilution of the virus-containing supernatants in DMEM for 2 h. The inoculum was removed, and cells washed and incubated with 200 μL DMEM containing 1 μg/mL TPCK-trypsin. After 48 h of incubation, the cytopathic effect was scored microscopically, and the TCID_50_ dose was calculated according to the Reed–Muench methods.

### Immunofluorescence Assay (IFA)

Cells from each group were fixed with 4% paraformaldehyde for 30 min, and then were permeabilized with 0.1% Triton-X100 for 5 min. After blocking with 2% bovine serum albumin for 20 min, they stained with a rabbit polyclonal antibody against NP (GTX125989) or a mouse Monoclonal antibody against MFN2 (ab56889, Abcam, United States) in 1:100 dilution at 4°C for 12 h. The secondary antibodies conjugated with Alexa Fluor 488 anti-mouse IgG in 1:200 dilution or 555 conjugated goat-rabbit IgG (Life Technology, NY, United States) in 1:200 dilution were applied for 45 min at room temperature. Nuclei were counterstained with DAPI (Beyotime, Shanghai, China), and the cells were visualized and analyzed using a confocal laser scanning microscope.

### Lung Histopathology and Virus Titers

Lungs from each group of mice after 5 dpi were removed and immediately fixed in 4% buffered formalin. Subsequently, the lung tissue was embedded in paraffin wax and 4 μm thick sections were sliced and stained with hematoxylin and eosin (H&E). For determination of virus titer in lung, lung tissues from euthanized mice were aseptically removed and homogenized in DMEM plus antibiotics to achieve 10% (w/v) suspensions, followed by centrifuged at 1400 *g* for 20 min at 4°C. TCID_50_ assay was performed to determine the infectivity of virus in the supernatant as described above.

### EGFP-MFN2 Plasmid Transfection

A549 cells were cultured on 6-well plate (NEST Biotech) in 50000 cells/ml and subsequently prepared for EGFP-MFN2 (TransGen Biotech) transfection to reach 50–60% confluence. A non-targeting vector (TransGen Biotech) was used as negative control. The transfection, using Lipofectamine^®^ LTX & PLUS^TM^ Reagent (Invitrogen), was carried out as described in the instructions of the manufacturer. Forty eight hours after transfection, cells were infected with 10 TCID_50_ H1N1 for 2 h. Subsequently, cells were performed to western blotting analysis at 12 hpi.

### Western Blotting Analysis

For immunoblotting analysis, lung samples and cell lysates lysed by RIPA buffer (Beyotime, China) were resolved by SDS–PAGE and transferred to the polyvinylidene fluoride membrane (Millipore, United States). Immunoblots were visualized by the ECL system (Fdbio Science, China). The following antibodies were used in immunoblotting analysis: antibodies for anti-IFITM3 (1:2,000), anti-MFN2 (1:2,000), anti-IFN-β (1:500) antibody and anti-IL-1β (1:1,000) were from Abcam. β-actin antibody (1:2,000) was purchased from Fude Biotechnology. Anti-phospho-IRF3 (1:1,000), and anti-TNF-α (1:2,000) antibodies were purchased from Cell Signaling Technology. Anti-MAVS (1:500) antibodies were obtained from Proteintech Group. β-actin was used as an internal control and the relative densities of the measured protein were quantified by image J software.

### Statistical Analysis

All data are expressed as mean ± standard deviation (SD) from at least three independent experiments. Differences during experiments were analyzed by unpaired one-way ANOVA (Tukey test) of the GraphPad Prism 5 system. Kinetics of mortality and morbidity are analyzed by Kaplan–Meier curves and log-rank test with Bonferroni adjustment. A value of *P* < 0.05 was defined as statistically significant.

## Results

### Protective Effects of Epigoitrin on Influenza Infection in Restraint-Stress Mice

**FIGURE 1:**
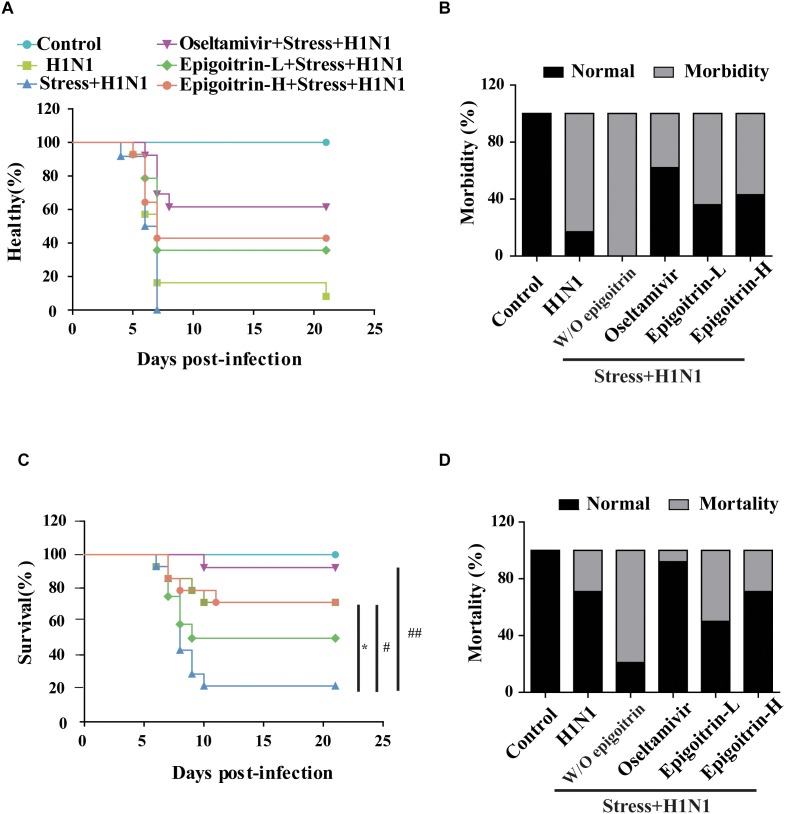
Epigoitrin attenuated the morbidity and mortality caused by influenza infection in stressed mice. **(A,B)** Healthy curve and morbidity caused by influenza infection in restraint mice. **(C,D)** Survival curve and mortality caused by influenza infection in restraint mice. “W/O epigoitrin” indicates without epigoitrin treatment. Epigoitrin-H and Epigoitrin-L, respectively, represent the higher dose of epigoitrin (176 mg/kg/d) and the lower dose of epigoitrin (88 mg/kg/d). The difference was considered statistically significant at ^∗^*P* < 0.05 vs. H1N1 group; ^#^*P* < 0.05 and ^##^*P* < 0.01 vs. “Stress+H1N1” group. Data were obtained from 10–14 animals in each group.

To establish a susceptible animal model, mice were loaded with restraint stress for 22 h before H1N1 virus challenge were employed. The experimental mice were monitored daily for 21 days. Compared with “H1N1” group, the emergence of clinical symptoms, including lack of appetite, inactivity, ruffled fur, a hunched posture and respiratory distress had an earlier tendency in “Restraint stress + H1N1” group (Figure 1A). Moreover, the morbidity of “Restraint stress + H1N1” group increased to 100% (Figure 1B). These results suggested that restraint stress could exacerbate clinical development of influenza disease and make the host susceptible to the influenza virus. Based on this model, the protective effects of epigoitrin against H1N1 virus in mice were investigated. As shown in Figure 1C,D, mice in the “Restraint stress + H1N1” group had a higher morbidity rate (100% vs. 83%) and lower mean time to sickness (6.33 ± 0.89 vs. 8.43 ± 5.36 days) compared to the “H1N1” alone group. Likewise, the survival rate of “Restraint stress + H1N1” group decreased from 71 to 50.0% compared with the “H1N1” alone group (*P* < 0.01), and the mean day to death (MDD) decreased from 17.29 ± 6.16 to 10.86 ± 5.7 days (*P* < 0.05). However, the administration of epigoitrin at 176, 88 mg/kg/d saved 50 and 29% of “Restraint stress + H1N1” group, respectively, and prolonged MDD with a lower morbidity sign. As a positive control, oseltamivir remarkable decreased the morbidity rate to 38% and markedly raised the survival rate to 92%. These results demonstrated that epigoitrin treatment effectively increased survival rate and protected mice from lethal infection with influenza.

### Effects of Epigoitrin on Influenza Infection in the Lung of Restraint-Stress Mice

**FIGURE 2:**
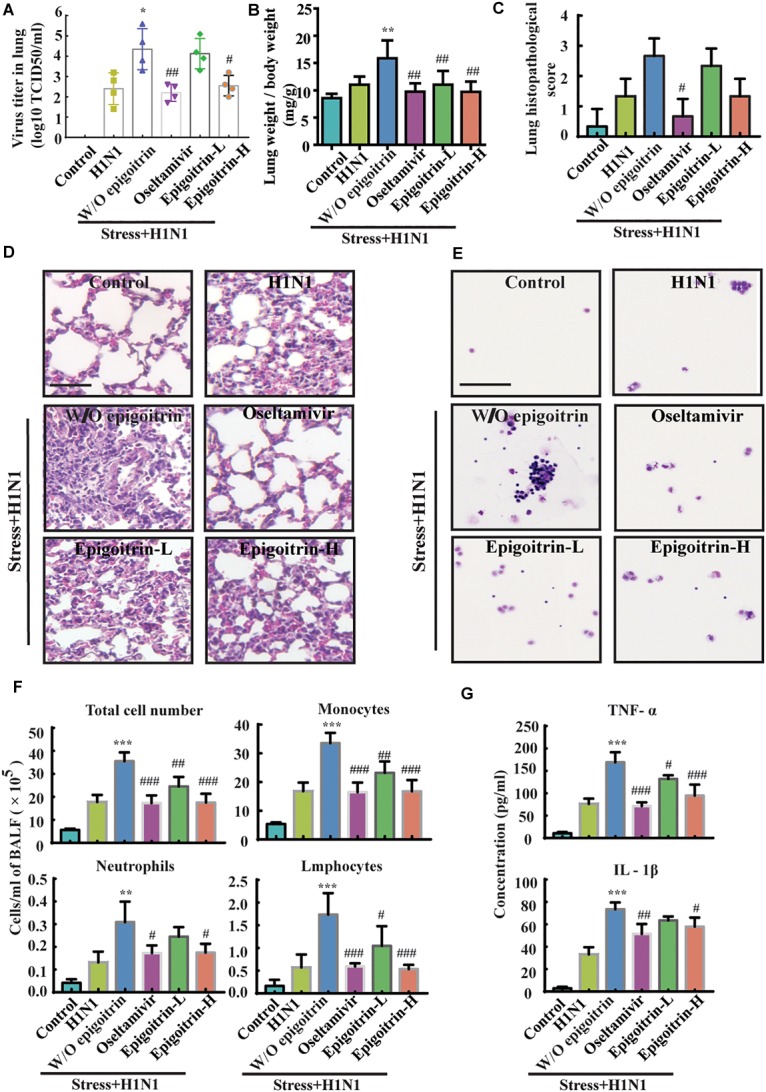
Epigoitrin protected against pneumonia caused by influenza infection in stressed mice. **(A,B)** The effects of epigoitrin on the viral titres in the lungs (*n* = 4) and the lung index (*n* = 6). **(C,D)** Lung pathological scores (*n* = 3) and histopathologic changes on the 5th day after influenza virus challenge, stained by H&E (scale bar = 50 μm). **(E,F)** Effects of epigoitrin on the changes of types of infiltrated inflammatory cells and cell numbers in BALF (scale bar = 100 μm). **(G)** Effects of epigoitrin on the levels of TNF-α, IL-1β in BALF (*n* = 4). The difference was considered statistically significant at ^∗^*P* < 0.05, ^∗∗^*P* < 0.01, ^∗∗∗^*P* < 0.001 vs. H1N1 group; ^#^*P* < 0.05, ^##^*P* < 0.01, ^###^*P* < 0.001 vs. “Stress+H1N1” group.

To further evaluate the therapeutic efficacy of epigoitrin against influenza infection in stressed mice, virus titers in the lung were measured after 5 dpi. No virus was detected in lung tissues of the Control group. The virus titer in “Restraint stress + H1N1” group was significantly higher than that in “H1N1” alone group (4.35 ± 0.50 vs. 2.40 ± 0.39 Log10 TCID_50_/ml), whereas virus titer were 2.55 ± 0.25 Log10 TCID_50_/ml and 4.13 ± 0.37 Log10 TCID_50_/ml in the high- and low-dose epigoitrin-treated groups (Figure 2A), respectively. H1N1 virus titers in stressed mice after epigoitrin treatment markedly decreased. Histopathological examination by H&E staining was performed to investigate pathological changes after virus infection. As shown in Figure 2C, mild inflammatory cell infiltration was observed in the lungs of mice infected H1N1 virus. More severe inflammatory cell infiltration in the interstitium and alveoli (Figure 2D) were found in the “Restraint stress +H1N1” group. Meanwhile, a higher lung index and histopathological score presented in the “Restraint stress + H1N1” group (Figure 2B,C), compared to the “Virus” alone group. In contrast, treatment with oseltamivir, or epigoitrin-H significantly reduced the numbers of total cell and infiltration of neutrophils, monocytes and lymphocytes in BALF (Figure 2E,F) with a lower index and histopathological score. Moreover, epigoitrin treatment effectively lowered TNF-α and IL-1β levels in BALF from “Restraint stress + H1N1” group (Figure 2G).

### Epigoitrin Improved the MAVS Antiviral Signaling Pathway in Restraint Stressed Mice

**FIGURE 3:**
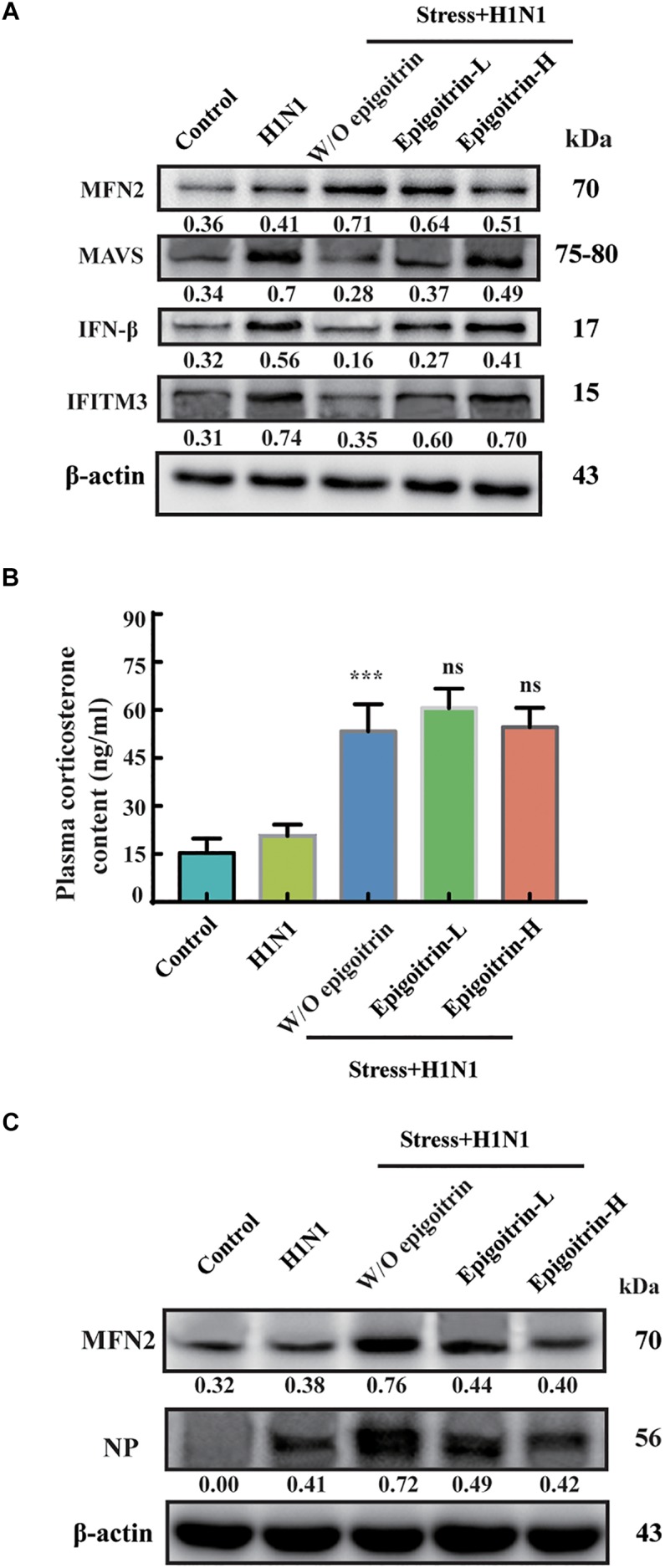
Epigoitrin improved MAVS antiviral signaling after influenza infection in stressed mice. **(A)** Effects of epigoitrin on MFN2, MAVS, IFN-β, and IFITM3 protein expressions in the lung tissues. **(B)** Effects of epigoitrin on corticosterone level in the plasma. **(C)** Effects of epigoitrin on MFN2, NP protein expressions in the lung tissues. Actin was used as an internal control and the relative densities of the measured protein were quantified by image J software. ^∗∗∗^*P* < 0.001 vs. H1N1 group; ns, no significance (*P* ≥ 0.05) vs. Stress+H1N1 group.

Previous studies had suggested that restraint stress-induced influenza viral susceptibility was closely related to innate immunity (Glaser and Kiecolt-Glaser, 2005). After infection, H1N1 virus mRNA was recognized by RIG-I/MAVS/IRF3 pathway and induced type I IFNs secretion, which could suppress viral replication, boost adaptive immunity, and limit acute lung injury (Arimori et al., 2013; McNab et al., 2015). To confirm that the differences in virus pathogenicity between the “Restraint stress + Virus” and epigoitrin groups were due to an improvement in type I IFNs pathway, the expression levels of protein related to this pathway were evaluated. The protein expressions of MAVS, IFN-β and IFITM3 were assayed in the lung tissue. Large increases in the protein expressions of MAVS, IFN-β, and IFITM3 were observed upon viral infection. Compared to the “H1N1” group, restraint stress obviously reduced these protein expressions with a higher MFN2 level. However, this process was improved by epigoitrin treatment (Figure 3A). Stressors could activate the hypothalamic-pituitary-adrenal axis and thereby trigger increases in stress hormone levels, which lead to dysregulation of immune function. Our previous study revealed that restraint stress-induced influenza viral susceptibility was specifically associated with corticosterone secretion (Chen et al., 2017). Hence, effects of epigoitrin on corticosterone level in the plasma of stressed mice were investigated. As show in Figure 3B,C, restraint stress significantly elevated the plasma corticosterone levels and the expression of MFN2 and virus nucleoprotein (NP) in lungs of mice. Interestingly, Epigoitrin-H and Epigoitrin-L had no significant effects on the plasma corticosterone level, but decreased the protein level of MFN2 and NP in stressed mice. It could be inferred that restraint stress-induced influenza viral susceptibility might be associated with corticosterone secretion. Epigoitrin treatment would boost resistance to influenza through the mitochondrial antiviral signaling pathway independent of inhibiting corticosterone secretion.

### Epigoitrin Reduced H1N1 Viral Expression in Corticosterone-Loaded A549 Cell

**FIGURE 4:**
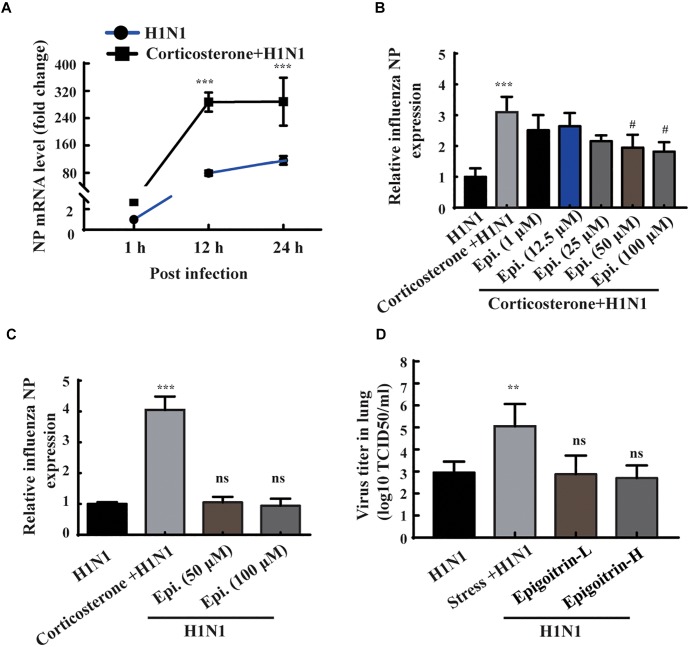
Epigoitrin reduced H1N1 viral expression in corticosterone-loaded A549 cell. **(A)** A549 cells were treated with or without corticosterone (100 μM) for 48 h before viral infection and the NP gene expression were measured by RT-qPCR at the indicated time. **(B,C)** Effects of epigoitrin on the NP gene expression in H1N1-infected A549 cell pretreated with or without corticosterone. **(D)** Effects of epigoitrin on the viral titers of lungs in stressed or unstressed mice (*n* = 4). The difference was considered statistically significant at ^∗∗^*P* < 0.01, ^∗∗∗^*P* < 0.001 vs. H1N1 group; ^#^*P* < 0.05 vs. corticosterone +H1N1 group; ns, no significance (*P* ≥ 0.05) vs. H1N1 group.

According to the results of animal experiments (Figure 3B), restraint stress-induced influenza viral susceptibility was associated with corticosterone secretion. Thus, corticosterone was utilized to establish a stress-induced susceptibility model *in vitro*. The gene expression of NP was assessed to reflect the viral replication level. As shown in Figure 4A, the intracellular viral abundance in the cells treated with corticosterone and H1N1 was significantly higher than that in cells only treated with virus at 12 and 24 hpi. The susceptibility to H1N1 infection caused by corticosterone is independent of viral entry, because there was no statistically significant difference in NP gene expression between the two groups level at 1 hpi. Based on this model, we examined the antiviral effects of epigoitrin. A dose-dependent inhibition in the mRNA expression of NP was observed in cells pretreated by epigoitrin compared with levels in untreated cells after corticosterone load (Figure 4B). However, this preventive effect of epigoitrin on NP gene expression in H1N1 susceptibility model may be not due to its direct antiviral ability, because epigoitrin pretreatment had no significantly inhibitory effect on the expression of viral gene NP in A549 cell (Figure 4C) and virus titer in the lungs of unstressed mice after H1N1 infection (Figure 4D). These result were consistent with the fact that pre-treatment with epigoitrin didn’t exert a prophylaxis effect on H1N1 infection (Xiao et al., 2016).

### Epigoitrin Inhibited H1N1 Replication Through Promoting IFN-β Generation in Corticosterone-Induced Viral Susceptibility Model

**FIGURE 5:**
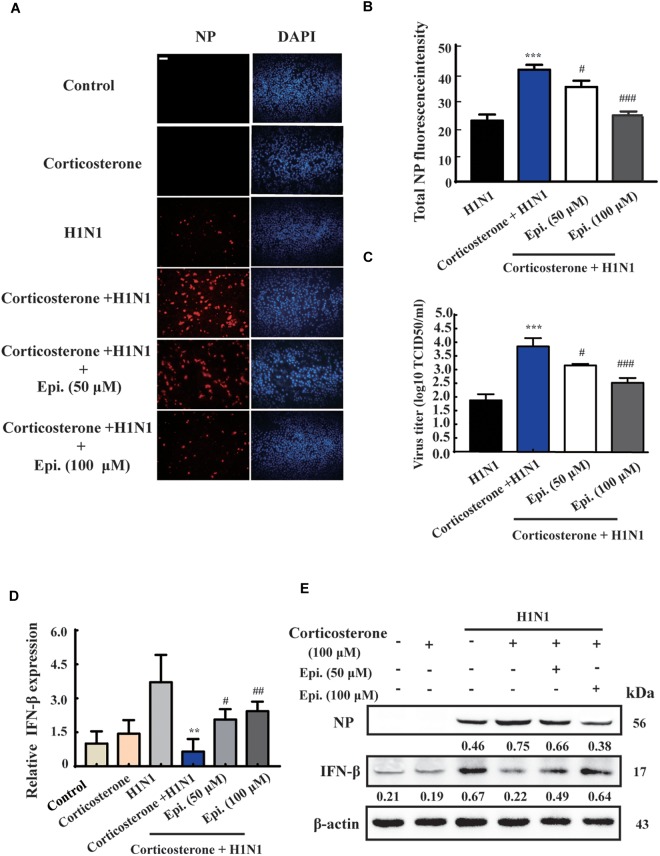
Epigoitrin inhibited H1N1 replication and promoted IFN-β generation after influenza infection in stress cell model. **(A)** Cells stained for influenza A virus NP at 12 hpi (red) and the cell nuclei were stained by DAPI (blue). Bar = 50 μm. **(B)** The total fluorescent intensity was determined to reflect the levels of NP. **(C)** Related infectious viral titer was detected by TCID_50_ assay. **(D,E)** Effects of epigoitrin on IFN-β gene and protein expression were analyzed by qRT-PCR and Western blotting. The difference was considered statistically significant at ^∗∗^*P* < 0.01, ^∗∗∗^*P* < 0.001 vs. H1N1 group; ^#^*P* < 0.05, ^##^*P* < 0.01, ^###^*P* < 0.001 vs. corticosterone +H1N1 group.

To further investigate the protective effect of epigoitrin on reducing influenza susceptibility *in vitro*, NP protein levels and virus titers were measured. As shown in Figure 5A, no immunofluorescence of viral NP was found in the “Control” and “Corticosterone” group. A significant increase of red fluorescence intensity (Figure 5B) and virus titer (Figure 5C) were observed in H1N1-infected cells with corticosterone pretreatment, which could be attenuated by epigoitrin treatment. Moreover, in cells pretreated with corticosterone, the expression levels of H1N1-triggered IFN-β gene (Figure 5D) and protein (Figure 5E) were significantly reduced, while epigoitrin treatment improved their production and reduced viral NP protein.

### MFN2 Was Engaged in the Regulation of IFN-β Production by Epigoitrin in Corticosterone-Induced Viral Susceptibility Model

**FIGURE 6:**
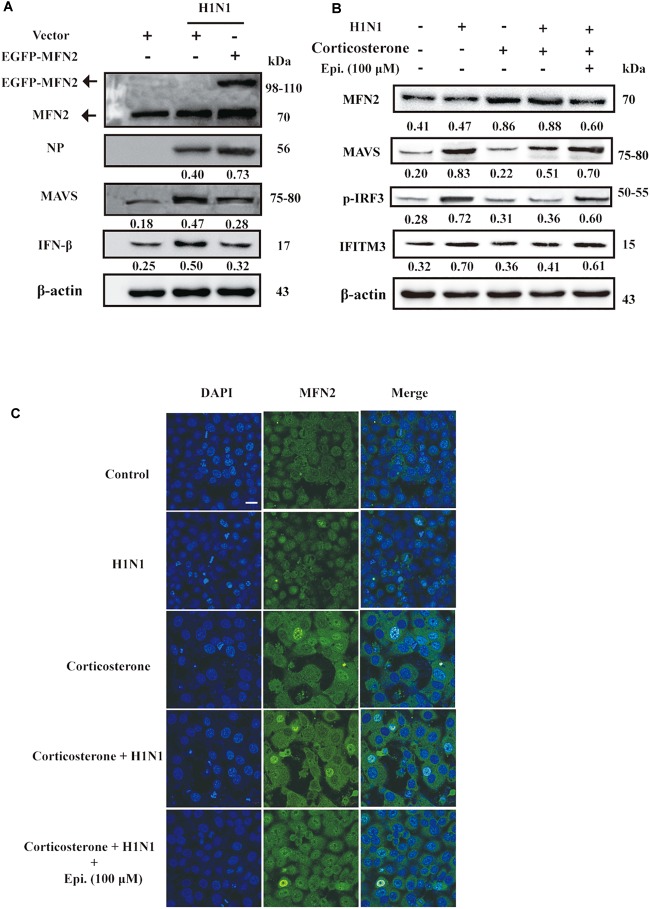
MFN2 was involved in the regulation of IFN-β production by epigoitrin in stress cell model. **(A)** Effects of EGFP-MFN2 overexpression on H1N1 infection. **(B)** The levels of protein expression by the MFN2, MAVS, p-IRF3, and IFITM3 gene relative to the level of β-actin expression were determined using the respective antibodies. **(C)** Representative fluorescence images showing the levels of MFN2 protein expression. Bar = 20 μm.

The antiviral RIG-I-like Receptor (RLR) signaling played a key role for IFN-β production in the mammalian immune response during H1N1 infection. Accumulated findings had unveiled that mitochondrial dynamics participated in RLR signaling transduction, functioning as signaling platforms and contributing to effector responses (West et al., 2011). MFN2, a mitofusin protein, had been shown to interact with MAVS and suppressed MAVS activating the IFN-β generation (Yasukawa et al., 2009). To examine the effects of MFN2 overexpression on H1N1 infection, we transfected cells with EGFP-MFN2 for western blot analysis. H1N1-infected cells showed higher IFN-β, an indicator of RLR signaling activation, and lower NP production when compared to cells transfected with MFN2. Moreover, higher MAVS expression was also noted in H1N1-infected cells without MFN2 transfection while lower antiviral activity appeared in the MFN2-overexpressing cells (Figure 6A). Hence, to explore whether epigoitrin could down-regulate the level of MFN2 protein and improved the MAVS antiviral signaling in H1N1 susceptibility model. Increased MFN2 protein was detected in both “Corticosterone” and “Corticosterone +Virus” groups, which resulted in a decreased level of the MAVS antiviral signaling-related proteins, MAVS, p-IRF3, and IFITM3. However, epigoitrin treatment could reduce the MFN2 protein expression and improve MAVS antiviral signaling-related proteins production (Figure 6B). We also determined the effect of epigoitrin on MFN2 expression by immunofluorescence and the results were coincided with that from western blotting (Figure 6C).

## Discussion

Previously, we had utilized restraint stress, a commonly used stressor to establish mouse H1N1 susceptibility model and evaluate the anti-viral effect of medicines and compounds. Based on this model the protective effect of epigoitrin on influenza susceptibility *in vivo* was evaluated. Our results showed that substantially increased morbidity and mortality in restraint stress animals challenged with H1N1 virus. Moreover, restraint stress also significantly increased the virus titer and induced excessive production of pro-inflammatory cytokines such as IL-1β, TNF-α, which aggravated pathological changes of the lung tissues in H1N1-infected mice. In comparison, treatment with epigoitrin prior to infection led to improvement of these pathological indicators and reduced the risk of influenza virus infection in restraint-stressed mice.

The principal peripheral effectors of the stress system are glucocorticoids, which are regulated by the hypothalamic–pituitary–adrenal axis (Chrousos, 2009). Studies indicated that restraint stress-induced influenza viral susceptibility was associated with corticosterone secretion (Konstantinos and Sheridan, 2001; Chen et al., 2017). Thus, we utilized corticosterone to establish an A549 cell stress model, and evaluated the preventive effects of epigoitrin on H1N1 infection. In the present study, we found that corticosterone *in vitro* could disrupt the interferon innate immune pathways and increased influenza viral susceptibility, which eventually facilitated its own replication in host cells. This effect of corticosterone-induced immunosuppression on virus infection is improved by epigoitrin. Epigoitrin treatment could improve the MAVS antiviral signaling and promoted the generation of IFN-β in corticosterone-loaded A549 cells and stressed mice following H1N1 infection.

**FIGURE 7:**
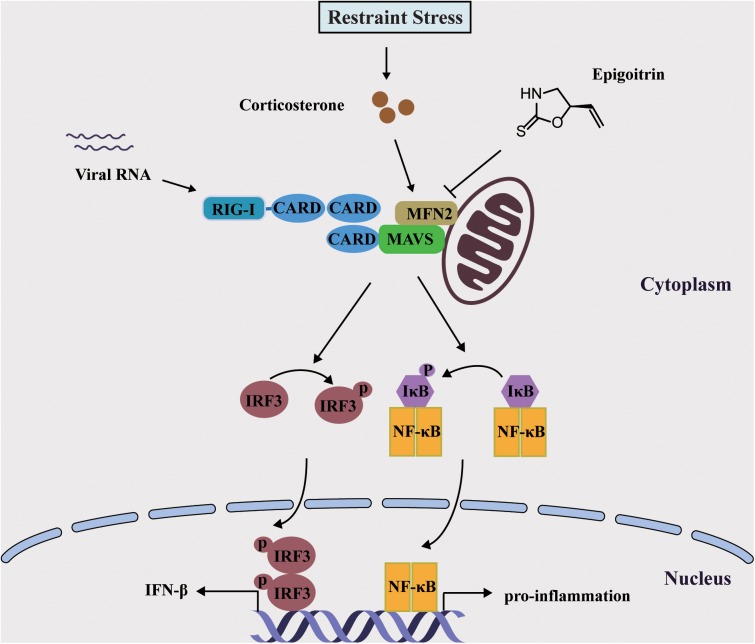
Schematic diagram of the mechanism of Epigoitrin-induced attenuation of H1N1 pathogenesis in the susceptible model.

Mitochondria were essential for triggering the cellular innate immune responses via MAVS against invading viruses, especially RNA viruses. This subsequently activated a signaling cascade that resulted in the phosphorylation and nuclear translocation of IRF3, leading to the expression of type I IFN (Iwasaki and Pillai, 2014). MFN2 acted as an inhibitor in regulating MAVS signaling independent of its function in mitochondrial fusion (Yasukawa et al., 2009). In accordance with this report, we also observed that overexpressed MFN2 dampened the generation of IFN-β following viral infection and increased H1N1 replication. The expression of MFN2 was considerably increased under restraint stress and corticosterone load, which was reduced by epigoitrin to improve the activation of MAVS antiviral signaling. These data indicated that epigoitrin could improve the suppression of innate immunity by restraint stress via downregulating MFN2 expression (Figure 7). Nevertheless, we do not know the underlying mechanism, further studies are required to investigate it.

In summary, our results demonstrated that epigoitrin reduced the susceptibility to H1N1 virus and the production of pro-inflammatory cytokines to alleviate pneumonia in restraint-stressed mice. Based on both the restraint-stressed mice model and corticosterone-loaded A549 cell model, epigoitrin was found to maintain MAVS antiviral signaling following H1N1 infection to ensure IFN-β production.

## Author Contributions

R-RH and Y-FL developed the study design and revised the manuscript. ZL participated in the study design, performed the experiments, analyzed the data, and wrote the manuscript. L-FL, X-HW, CJ, HC, D-HL, C-YY performed the experiments and analyzed the data. BL, F-QW, and HK participated in the study design and analyzed the data. All authors have read and approved the final version of the manuscript.

## Conflict of Interest Statement

The authors declare that the research was conducted in the absence of any commercial or financial relationships that could be construed as a potential conflict of interest.
